# RII*β*‐PKA in GABAergic Neurons of Dorsal Median Hypothalamus Governs White Adipose Browning

**DOI:** 10.1002/advs.202205173

**Published:** 2022-12-18

**Authors:** Bingwei Wang, Miao Zhao, Zhijie Su, Baohua Jin, Xiaoning Yang, Chenyu Zhang, Bingbing Guo, Jiebo Li, Weili Hong, Jiarui Liu, Yun Zhao, Yujia Hou, Futing Lai, Wei Zhang, Lihua Qin, Weiguang Zhang, Jianyuan Luo, Ruimao Zheng

**Affiliations:** ^1^ Department of Anatomy Histology and Embryology School of Basic Medical Sciences Peking University Beijing 100191 P. R. China; ^2^ Department of Pharmacology Institution of Chinese Integrative Medicine Hebei Medical University Shijiazhuang 050017 P. R. China; ^3^ Institute of Medical Photonics Beijing Advanced Innovation Center for Biomedical Engineering School of Biological Science and Medical Engineering Beihang University Beijing 100191 P. R. China; ^4^ Department of Medical Genetics School of Basic Medical Sciences Peking University Beijing 100191 P. R. China; ^5^ Department of Biochemistry and Molecular Biology School of Basic Medical Sciences Peking University Beijing 100191 P. R. China; ^6^ Neuroscience Research Institute Key Laboratory for Neuroscience of Ministry of Education Key Laboratory for Neuroscience of National Health Commission of the People's Republic of China Peking University Beijing 100191 P. R. China

**Keywords:** dorsal median hypothalamus, GABAergic neurons, RIIβ‐PKA, white adipose browning

## Abstract

The RII*β* subunit of  cAMP‐dependent protein kinase A (PKA) is expressed in the brain and adipose tissue. RII*β*‐knockout mice show leanness and increased UCP1 in brown adipose tissue. The authors have previously reported that RII*β* reexpression in hypothalamic GABAergic neurons rescues the leanness. However, whether white adipose tissue (WAT) browning contributes to the leanness and whether RII*β*‐PKA in these neurons governs WAT browning are unknown. Here, this work reports that RII*β*‐KO mice exhibit a robust WAT browning. RII*β* reexpression in dorsal median hypothalamic GABAergic neurons (DMH GABAergic neurons) abrogates WAT browning. Single‐cell sequencing, transcriptome sequencing, and electrophysiological studies show increased GABAergic activity in DMH GABAergic neurons of RII*β*‐KO mice. Activation of DMH GABAergic neurons or inhibition of PKA in these neurons elicits WAT browning and thus lowers body weight. These findings reveal that RII*β*‐PKA in DMH GABAergic neurons regulates WAT browning. Targeting RII*β*‐PKA in DMH GABAergic neurons may offer a clinically useful way to promote WAT browning for treating obesity and other metabolic disorders.

## Graphical abstract

The cAMP‐dependent protein kinase A (PKA) in GABAergic neurons in dorsal median hypothalamus (DMH) predominantly regulates white adipose browning. The enzymatic subtype‐conversion of PKA induced by RII*β* gene deficiency may be a crucial event for eliciting adipose browning. Targeting RII*β*‐PKA in DMH GABAergic neurons may offer a clinically useful way to promote adipose browning for treating obesity and metabolic disorders.

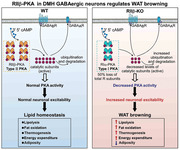

## 1 Introduction

PKA is a key mediator of signal transduction downstream of G protein‐coupled receptors in response to neurotransmitters and hormones.^[^ ^1^ ^]^ PKA plays a major role in the control of energy balance and metabolic homeostasis.^[^ ^2^ ^]^ PKA holoenzyme comprises two homodimeric regulatory subunits (R subunits) and two catalytic subunits (C subunits).^[^ ^3^ ^]^ In mice, there are four isoforms of R subunits (RI*α*, RI*β*, RII*α*, and RII*β*) and two isoforms of C subunits (C*α* and C*β*), expressed in tissue‐specific patterns.^[^ ^3a,b^ ^]^ PKA holoenzyme is inactive.^[^ ^4^ ^]^ The binding of cAMP to the R subunits leads to the release of active C subunits.^[^ ^5^ ^]^ The R subunits act as intrinsic inhibitors of the C subunits and also protect the C subunits from degradation.^[^ ^6^ ^]^ Notably, the RII*β* subunit is selectively expressed in three tissues known to regulate energy homeostasis: brain, WAT and brown adipose tissue (BAT), with limited expression elsewhere.^[^ ^2^, ^3^, ^4^, ^7^ ^]^ It has been reported that RII*β* knockout (RII*β*‐KO) mice exhibit lean phenotypes: a 50% decrease in WAT and a 10% reduction in body weight.^[^ ^3^, ^8^ ^]^ RII*β*‐KO mice also show increased WAT browning, elevated EE and are resistant to high‐fat diet‐induced obesity, fatty liver, dyslipidemia and diabetes.^[^ ^3^, ^8^, ^9^ ^]^ RII*β*‐KO mice have normal food intake and lengthened healthy life span.^[^ ^3^, ^8^, ^9^ ^]^ We observed that the level of uncoupling protein 1 (UCP1), the canonical marker of the browning of WAT, is robustly increased in WATs of RII*β*‐KO mice; however, whether WAT browning contributes to the healthy lean phenotypes of RII*β*‐KO mice is unknown.

The WAT browning has raised great interest because of its potential in counteracting obesity.^[^ ^10^ ^]^ The WAT browning is the result of the induction of beige adipocytes and/or the conversion of white adipocytes into beige adipocytes within WAT depots, leading to activated adipocytic mitochondria, enhanced UCP1 expression, reduced adipocyte volume, the formation of multilocular lipid droplets, and promoted fat utilization and thermogenesis, resulting in elevated EE and reduced adiposity. Stimulation of WAT browning reduces adiposity, improves lipid/glucose metabolism and whole‐body metabolic homeostasis, and extends lifespan.^[^ ^10^, ^11^ ^]^ The hypothalamus regulates the WAT browning via sympathetic nerves.^[^ ^12^ ^]^ The RII*β* subunit is highly expressed in the hypothalamus.^[^ ^2^ ^]^ Here, we show that RII*β*‐KO mice have typical adipose browning phenotypes. These observations suggest that the RII*β*‐KO mice could be an ideal model for deciphering the mechanism underlying the central regulation of adipose browning. Nevertheless, whether RII*β*‐PKA in hypothalamic neurons is linked to the regulation of WAT browning is still unclear.

The RII*β*‐expressing hypothalamic nuclei include arcuate nucleus (ARC), ventromedial hypothalamus (VMH), paraventricular hypothalamus (PVH), and dorsomedial hypothalamus (DMH).^[^ ^2^ ^]^ We previously reported that the reexpression of the RII*β* subunit in hypothalamic GABAergic neurons rescues the lean and metabolic phenotypes of RII*β*‐KO mice.^[^ ^2b^ ^]^ Reexpression of RII*β* subunit in ARC, VMH, or PVH did not affect the phenotypes.^[^ ^2b^ ^]^ These findings raised the possibility that the hypothalamic GABAergic neurons outside ARC, VMH, and PVH may be responsible for the lean and WAT browning phenotypes of RII*β*‐KO mice. Notably, the GABAergic neurons are the dominant neuronal population in DMH.^[^ ^13^ ^]^ The DMH, an autonomic center and a master driver of activity of the sympathetic nerve system (SNS), is involved in the regulation of WAT browning and metabolic health.^[^ ^12^, ^14^ ^]^ DMH‐specific deletion of Gq/11alpha reduces sympathetic nerve activity, decreases EE and adipocytic UCP1, and causes obesity.^[^ ^15^ ^]^ Knockdown of glucagon‐like peptide‐1 receptor in the DMH decreases EE and adipocytic UCP1, and increases adiposity and body weight.^[^ ^16^ ^]^ Knockdown of NPY in the DMH GABAergic neurons promotes WAT browning, leading to increased heat production and reduced adiposity.^[^ ^17^ ^]^ Nevertheless, whether DMH GABAergic neurons are involved in the regulation of WAT browning is unclear. Whether RII*β*‐PKA in DMH GABAergic neurons is a key factor contributing to the generation of the adipose browning phenotypes of RII*β*‐KO mice is unknown.

To investigate the potential role of RII*β*‐PKA in the DMH GABAergic neurons for the regulation of WAT browning, a series of genetically modified mouse strains, single‐cell sequencing (scRNA‐seq), transcriptome sequencing (RNA‐seq), whole‐cell patch‐clamp technique, designer receptors exclusively activated by designer drugs (DREADDs), optogenetics, and neuropharmacological approach were employed in this study. Our findings reveal that RII*β*‐PKA in DMH GABAergic neurons may serve as an important neuronal population for controlling WAT browning, and provide a novel insight into the mechanism of the central regulation of whole‐body metabolic homeostasis.

## 2 Results

### 2.1 RII*β*‐KO Mice Exhibit Increased WAT Browning

**Figure 1:**
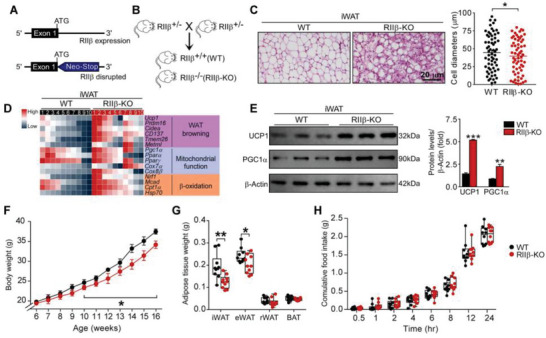
RII*β*‐KO mice exhibit increased WAT browning. A) Schematic of the knockout strategy for RII*β* gene. B) Breeding strategy for generation of RII*β* ^+/+^ mice (WT) and RII*β* ^−/−^ mice (RII*β*‐KO). C) Representative images of hematoxylin and eosin (H&E) staining of iWAT and the size profiling of adipocytes from iWAT. Scale bar indicates 20 µm. D) Heatmap shows mRNA levels of the genes associated with WAT browning in iWAT. E) Representative immunoblots of UCP1, PGC1*α* and *β*‐Actin from iWAT, and the quantified ratio of UCP1/*β*‐Actin and PGC1*α*/*β*‐Actin. F) Body weight. G) Fat‐pad weight. H) Cumulative food intake and total food intake. WT *n* = 10; RII*β*‐KO *n* = 10. Values show mean ± SEM. Student's *t*‐test was used for analysis of the data in (C), (E), (G), and (H). One‐way ANOVA with Tukey's post hoc test was used for analysis of the data in (F). *p < 0.05.

To determine whether the deficiency of RII*β* induces WAT browning, the metrics of the adipose browning were applied. The strategy for RII*β* gene knockout and breeding strategy to generate RII*β* ^−/−^ mice (RII*β*‐KO) mice were illustrated in **Figure** **1**A,B. The size of adipocytes in iWAT of RII*β*‐KO mice showed about 33.00% smaller than that of controls and with much more abundant multilocular lipid droplets (LDs) (Figure 1C). A sustained expression of the adipose browning‐associated genes, including *Ucp1*, *Prdm16*, *Cidea*, *CD137*, *Tmem26*, and *Metrnl*; the mitochondrial function‐related genes, including *Pgc1*α, *PPAR* *α*, *Cox7* *α* *1*, *Cox8* *β*, *Nrf1*, *Mcad*, *Cpt1* *α*, and *HSP70* were increased in iWAT of RII*β*‐KO mice (Figure 1D). The protein levels of UCP1, a canonical marker of WAT browning; and PGC1*α*, a key regulator for mitochondrial biogenesis, were increased compared with controls (Figure 1E). The body weight of RII*β*‐KO mice (final weight mean ± SEM: WT, 37.5 ± 0.1 g; RII*β*‐KO, 34.2 ± 0.2 g) were lower than that of controls (Figure 1F). The weight of inguinal WAT (iWAT) (weight mean ± SEM: WT, 0.18 ± 0.05 g; RII*β*‐KO, 0.13 ± 0.02 g) and epididymal WAT (eWAT) (weight mean ± SEM: WT, 0.23 ± 0.04 g; RII*β*‐KO, 0.19 ± 0.01 g) were decreased in RII*β*‐KO mice; whereas the weights of retroperitoneal WAT (rWAT) or BAT were not changed (Figure 1G). We observed that the expression of genes associated with WAT browning were markedly increased in eWAT and BAT of RII*β*‐KO mice, while a slight but not significant increase in rWAT (Figure S1A,C,E, Supporting Information). Moreover, the protein levels of UCP1, and TH (the canonical marker of sympathetic activity) were also increased in eWAT and BAT (Figure S1B,D,F, Supporting Information). Consistent with our previous reports,^[^ ^2b^ ^]^ the cumulative food‐intake did not differ between RII*β*‐KO and control mice (Figure 1H). Together, these observations demonstrate that RII*β*‐KO mice have a remarkable adipose browning phenotype accompanied with lower body weight and fat mass, and determine that WAT browning is one of the metabolic phenotypes of RII*β*‐KO mice.

### 2.2 Reexpression of RII*β* in DMH GABAergic Neurons Abrogates WAT Browning

**Figure 2:**
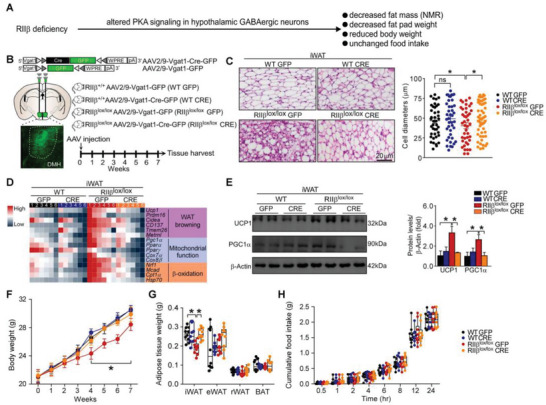
Reexpression of RII*β* in DMH GABAergic neurons abrogates WAT browning. A) RII*β* deficiency in hypothalamic GABAergic neurons leads to leanness and other healthy phenotypes. B) Schematic diagram of the experiment and representative fluorescent image showing injection of AAV into the DMH. C) Representative images of H&E staining of iWAT and the size profiling of adipocytes from iWAT. Scale bar indicates 20 µm. D) Heatmap shows mRNA levels of the WAT browning associated genes in iWAT (WT GFP *n* = 6; WT CRE *n* = 6; RII*β* ^lox/lox^ GFP *n* = 6; RII*β* ^lox/lox^ CRE *n* = 6). E) Representative immunoblots of UC1, PGC1*α*, and *β*‐Actin from iWAT, and the quantified ratio of UCP1/*β*‐Actin, PGC1*α*/*β*‐Actin. F) Body weight. G) Fat‐pad weight. H) Cumulative food intake and total food intake. WT GFP *n* = 10; WT CRE *n* = 10; RII*β* ^lox/lox^ GFP *n* = 10; RII*β* ^lox/lox^ CRE *n* = 10. Values show mean ± SEM. Two‐way ANOVA with Tukey's post hoc test was used for analysis of the data in (C) and (E)–(H). *p < 0.05.

We previously reported that RII*β* deficiency altered PKA signaling in hypothalamic GABAergic neurons and thus decreased fat mass and body weight (**Figure** **2**A); reexpression of the RII*β* subunit in these neurons rescued the lean phenotype of RII*β*‐KO mice; whereas, reexpression of the RII*β* subunit in ARC, VMH or PVH did not affect the phenotypes.^[^ ^2b^ ^]^ These observations raised an intriguing possibility that the hypothalamic GABAergic neurons may account for the metabolic phenotypes of RII*β*‐KO mice. Moreover, these findings pointed out that RII*β*‐PKA in the GABAergic neurons of the DMH may be linked to the generation of the metabolic phenotypes of RII*β*‐KO mice. As described previously,^[^ ^2b^ ^]^ a loxP‐flanked cassette containing the neomycin resistance gene was inserted between the transcription start site and the ATG codon of the RII*β* gene; mice carrying the Cre recombinase transgene can delete the neo‐STOP sequence, and thus RII*β* gene is reexpressed in the Cre‐expressing cells. Mice homozygous for the inactive allele, designated as RII*β* ^lox/lox^, do not express RII*β* protein and are phenotypically identical to the RII*β*‐KO mice. Thus, both RΙΙ*β* ^lox/lox^ and RΙΙ*β* ^lox/−^ mice are referred to as RII*β*‐KO mice, and RΙΙ*β* ^lox/+^ mice are referred to as RII*β* ^HET^ mice. In this study, using the Vgat‐ires‐Cre mouse line, we specifically reexpressed RII*β* in hypothalamic GABAergic neurons (RII*β* ^Vgat‐CRE^ mice) to test whether these neurons may contribute to the adipose browning phenotypes of RII*β*‐KO mice (Figure S2A,B, Supporting Information). We found that RII*β* subunit reexpression in the hypothalamic GABAergic neurons normalized the typical adipocytic morphology of adipose browning, the expression levels of the browning‐associated genes, and the protein levels of UCP1 and PGC1*α* (Figure S2C–E, Supporting Information). Reexpression of RII*β* subunit in the hypothalamic GABAergic neurons restored the body weight (final weight mean ± SEM: RII*β* ^HET^, 24.0 ± 0.6 g; RII*β* ^lox/−^, 22.7 ± 0.5 g; RII*β* ^Vgat‐CRE^, 23.0 ± 0.9 g) and adiposity (iWAT weight mean ± SEM: RII*β* ^HET^, 0.21 ± 0.02 g; RII*β* ^lox/−^, 0.12 ± 0.01 g; RII*β* ^Vgat‐CRE^, 0.22 ± 0.02 g) (Figure S2F,G, Supporting Information); without affecting the food intake (Figure S2H, Supporting Information).

To further determine whether the signaling via RII*β*‐PKA within DMH GABAergic neurons may play a role in the regulation of WAT browning, AAVs coding for Vgat1‐promoter‐driven Cre recombinase were stereotaxically injected into the DMH of RII*β* ^lox/lox^ mice to specifically reexpress RII*β* subunit in DMH GABAergic neurons of RII*β*‐KO mice (Figure 2B; Figures S3 and S4, Supporting Information). We observed that the adipose browning and metabolic phenotypes of RII*β*‐KO mice, including the decreased adipocytic size, increased multilocular lipid droplets (LDs) and enhanced expression levels of the adipose browning genes, were restored by reexpression of RII*β* in DMH GABAergic neurons (Figure 2C–E). Moreover, reexpression of RII*β* subunit in DMH GABAergic neurons restored the body weight (final weight mean ± SEM: RII*β* ^lox/lox^ GFP, 28.5 ± 0.9 g; RII*β* ^lox/lox^ CRE, 29.9 ± 1.0 g; WT GFP, 30.6 ± 0.4 g; WT CRE, 30.5 ± 0.6 g) and adiposity (iWAT weight mean ± SEM: RII*β* ^lox/lox^ GFP, 0.18 ± 0.01 g; RII*β* ^lox/lox^ CRE, 0.25 ± 0.01 g; WT GFP, 0.26 ± 0.01 g; WT CRE, 0.24 ± 0.02 g) (Figure 2F,G) without affecting the food intake (Figure 2H). Overall, these results suggest that RII*β*‐PKA in DMH GABAergic neurons may be tightly involved in the regulation of WAT browning; and the deficiency of RII*β* subunit of PKA in DMH GABAergic neurons may be the primary cause for generating the adipose browning phenotype of RII*β*‐KO mice.

### 2.3 Increased GABAergic Activity in DMH GABAergic Neurons of RII*β*‐KO Mice

**Figure 3:**
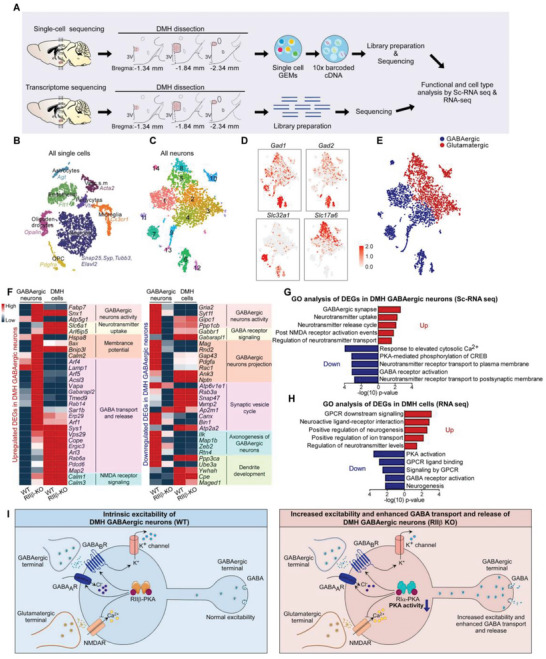
Increased GABAergic activity in DMH GABAergic neurons of RII*β*‐KO Mice. A) Schematic representation of the experimental workflow. sc‐RNAseq: WT *n* = 5; RII*β*‐KO *n* = 5. Bulk RNAseq: WT *n* = 5; RII*β*‐KO *n* = 5. B) tSNE visualization of eight transcriptionally distinct clusters expressing canonical markers. Astrocytes (*Agt*); endothelial cells (*Flt1*); microglia (*Cx3cr1*); neurons (*Snap25*, *Syp*, *Tubb3*, *Elavl2*); oligodendrocytes (*Opalin*); OPC, oligodendrocyte precursor cells (*Pdgfra*); pericytes (*Vtn*); vascular smooth muscle (*Acta2*). C) tSNE visualization of 13 transcriptionally distinct clusters of neurons after the first iteration of unsupervised clustering (*n*  =  12124 neurons). D) Normalized expression of Gad1, Gad2, Slc32a1, and Slc17a6 in each cell shown on t‐SNE plot after the second iteration of unsupervised clustering of neurons. E) Neurons were classified as either GABAergic or glutamatergic based on the expression of canonical markers (*n* = 12124 neurons). F) Heatmap shows mRNA levels of the GABA function associated genes in DMH at both single cell RNA sequencing (GABAergic neurons) and bulk RNA sequencing (DMH cells) resolution. G) GO analysis of DEGs in DMH GABAergic neurons (Sc‐RNA seq) between RII*β*‐KO and WT mice. H) GO analysis of DEGs in DMH GABAergic neurons (RNA seq) between RII*β*‐KO and WT mice. I) Diagram of the mechanisms underlying the increased excitability and enhanced GABAergic function of DMH GABAergic neurons in RII*β*‐KO mice.

The alteration of the PKA kinase system caused by the deficiency of RII*β* could account for the metabolic phenotypes of RII*β*‐KO mice. To decipher the molecular mechanisms by which RII*β*‐PKA in DMH GABAergic neurons may be linked to the regulation of adipose browning, we performed the genome‐wide single‐cell sequencing (scRNA‐Seq) and transcriptome sequencing (RNA‐Seq) analysis. We microdissected the DMH from fresh brain slices for RNA‐Seq, and processed single‐cell suspensions through the 10× Genomics Chromium Controller as previously described;^[^ ^18^ ^]^ and the microdissection was mapped for accuracy and reproducibility (**Figure** **3**A). Our initial database included the single‐cell expression data from RII*β*‐KO (*n* = 6369 cells) and WT (*n* = 5755 cells) samples. We assigned single cells to a given cell type defined by the known marker genes, including astrocytes (*Agt*), endothelial cells (*Flt1*), microglia (*Cx3cr1*), neurons (*Snap25*, *Syp*, *Tubb3*, *Elavl2*), oligodendrocytes (*Opalin*), oligodendrocyte precursor cells (*Pdgfra*), pericytes (*Vtn*), and vascular smooth muscle (*Acta2*),^[^ ^18^, ^19^ ^]^ enabling automated annotation of the cell types in the DMH (Figure 3B,C). We next used unsupervised, iterative clustering to distinguish molecularly distinct neuronal clusters. We found a dichotomy among DMH neuronal clusters based on the expression of the genes necessary for the synthesis and packaging of GABA and glutamate. In particular, Slc32a1, which encodes vesicular GABA transporter (Vgat), was expressed in the neuronal clusters of 1, 4, 6, 7, 9, 11, 12, 13, and 14. In contrast, another collection of clusters robustly expressed Slc17a6, which encodes vesicular glutamate transporter 2 (Vglut 2). The expression of the genes encoding synthetic enzymes for GABA [*Gad1*, which encodes GAD67; *Gad2*, which encodes GAD65] were largely tracked with Slc32a1^+^ clusters (Figure 3C–E). From this, we nominally categorized Slc32a1^+^ clusters as GABAergic and Slc17a6^+^ clusters as glutamatergic neurons (Figure 3E). Our previous studies and literatures revealed that GABAergic neurons and PKA activity are critically important in DMH for regulation of metabolic homeostasis.^[^ ^2^, ^8^, ^14^ ^]^ These studies contribute to form the criterion we used to select the differently expressed genes (DEGs). We performed a full set of bioinformatic strategy to analyze the transcriptomic changes. DEGs were identified using the DESeq2 bioconductor package, where genes differentially expressed over 1.5 folds between the groups, and those with adjusted *p* value ≤ 0.05 were considered as differentially expressed. DEGs associated with PKA activity (8 genes) and GABAergic function (39 genes) were observed. In addition, the neural function‐related DEGs in DMH of RIIß‐KO mice were also monitored and analyzed. Comparison between the RII*β*‐KO mice and WT mice further revealed that both up‐ and downregulated DEGs were highly cell‐type specific, with nearly all genes perturbed either in GABA transport and release or in membrane potential in DMH GABAergic neurons (Figure 3F). Similarly, DEGs in DMH glutamatergic neurons were related to glutamatergic activity (Figure S5A, Supporting Information).

To further decipher the molecular characteristics of GABAergic neurons in the DMH of RII*β*‐KO mice, GO analysis was performed. We found that the pathways associated with GABAergic activity, GABA transport and release, post NMDA receptor activation were induced (Figure 3G,H), while the pathways associated with PKA activity and GABA receptor were inhibited in RII*β*‐KO mice (Figure 3G,H). GO analysis of DEGs in DMH glutamatergic neurons shows that pathways associated with glutamatergic function were induced and the pathways associated with PKA activity were also inhibited (Figure S5B, Supporting Information).

To examine the neuronal excitability of GABAergic neurons in the DMH, the whole‐cell patch‐clamp technique was employed. AAV coding for Vgat1‐promoter‐driven green fluorescent protein (GFP) was stereotaxically injected into the DMH to specifically label the GABAergic neurons in the DMH (Figure S6A, Supporting Information). We found the frequency of action potentials (APs) in DMH GABAergic neurons of RII*β*‐KO mice were increased from 4.00 ± 0.57  Hz (cell‐attached patch‐clamp) and 4.48 ± 0.35 Hz (whole‐cell patch‐clamp) to 6.50 ± 0.61  Hz and 6.59 ± 0.59  Hz, respectively (Figure S6B,C, Supporting Information). The resting membrane potential was unchanged in RII*β*‐KO mice, as compared with controls (Figure S6D, Supporting Information). APs were elicited by a depolarizing current step of 40 pA. An increased number of APs in DMH GABAergic neurons were observed in RII*β*‐KO mice, from 16 ± 1 to 22 ± 1 (Figure S6E, Supporting Information). In addition, the mean input resistance of these neurons in RII*β*‐KO mice (938 ± 49 MΩ) was higher than that of WT mice (748 ± 47 MΩ) (Figure S6F, Supporting Information). The action potential threshold was unaltered in RII*β*‐KO mice (Figure S6G, Supporting Information). These results indicate that the neuronal activity of DMH GABAergic neurons is increased in the absence of the RII*β* subunit of PKA; and suggest that the increased excitability of DMH GABAergic neurons and the enhanced GABAergic transmission caused by the RII*β* deficiency may be the fundamental neurobiological processes underlying the adipose browning phenotypes of RII*β*‐KO mice.

Collectively, the single‐cell sequencing and transcriptome sequencing analyses indicate that the RII*β* deficiency may lead to increased excitability and enhanced GABA transport and release in DMH GABAergic neurons (Figure 3I).

### 2.4 Activation of DMH GABAergic Neurons Elicits Adipose Browning and Lowers Adiposity

**Figure 4:**
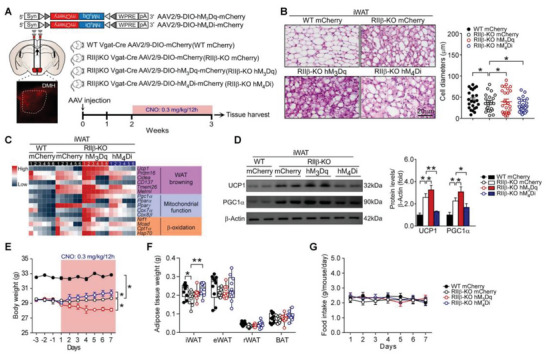
Activation of DMH GABAergic neurons elicits adipose browning and lowers adiposity. A) Schematic diagram of the experiment and representative fluorescent image showing injection of AAV into DMH. B) Representative images of H&E staining of iWAT and the size profiling of adipocytes from iWAT. Scale bar indicates 20 µm. C) Heatmap shows mRNA levels of the WAT browning associated genes in iWAT. WT GFP *n* = 6; RII*β*‐KO GFP *n* = 6; RII*β*‐KO hM_3_Dq *n* = 6; RII*β*‐KO hM_4_Di *n* = 6. D) Representative immunoblots of UCP1, PGC1*α*, and *β*‐Actin from iWAT, and the quantified ratio of UCP1/*β*‐Actin, PGC1*α*/*β*‐Actin. E) Body weight. F) Fat‐pad weight. G) Food intake. WT GFP *n* = 10; RII*β*‐KO GFP *n* = 10; RII*β*‐KO hM_3_Dq *n* = 10; RII*β*‐KO hM_4_Di *n* = 10. Values show mean ± SEM. Two‐way ANOVA with Tukey's post hoc test was used for analysis of the data in (B) and (E)–(H). *p < 0.05 and **p < 0.01.

Combining the electrophysiological analysis with the single‐cell sequencing and transcriptome sequencing analysis, we have determined the increased excitability of DMH GABAergic neurons in RII*β*‐KO mice. To further test whether the increased excitability of DMH GABAergic neurons may underlie the enhanced adipose browning, we employed a chemogenetic approach known as DREADDs. The Cre recombinase‐dependent AAV was bilaterally injected into the DMH of RII*β*‐KO‐Vgat‐Cre mice and also their WT littermates (so‐called WT GFP, RII*β*‐KO GFP, RII*β*‐KO hM_3_Dq, and RII*β*‐KO hM_4_Di, respectively), followed by treatment with Clozapine N‐oxide (CNO) (**Figure** **4**A). The AAV delivery sites are shown in Figure 4A, and GFP fluorescence was visualized. The activation of DMH GABAergic neurons by CNO induced a typical adipose browning morphology (Figure 4B), and increased expression levels of the canonical molecular markers associated with adipose browning, such as UCP1 and PGC1*α* (Figure 4C,D).

The activation of DMH GABAergic neurons reduced the body weight (final weight mean ± SEM: RII*β*‐KO GFP, 29.7 ± 0.1 g; RII*β*‐KO hM_3_Dq, 28.1 ± 0.2 g; RII*β*‐KO hM_4_Di, 30.4 ± 0.4 g; WT GFP, 32.8 ± 0.1 g) and the fat‐pad weight of iWAT (weight mean ± SEM: RII*β*‐KO GFP, 0.17 ± 0.01 g; RII*β*‐KO hM_3_Dq, 0.19 ± 0.01 g; RII*β*‐KO hM_4_Di, 0.22 ± 0.01 g; WT GFP, 0.21 ± 0.01 g) (Figure 4E,F). The CNO administration in mice did not affect food intake (Figure 4G). Conversely, the inhibition of DMH GABAergic neurons induced an opposite response (Figure 4B–F). In addition, similar results were observed in the optogenetic study (Figure S7A–G, Supporting Information). Taken together, these results suggest that DMH GABAergic neurons are involved in the central regulation of WAT browning; and the increased excitability of DMH GABAergic neurons underlies the adipose browning phenotypes of RII*β*‐KO mice.

### 2.5 Decreased PKA Activity in DMH GABAergic Neurons of RII*β*‐KO Mice

**Figure 5:**
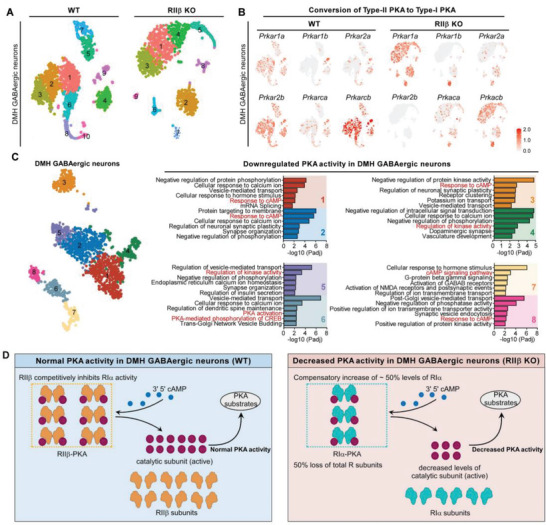
Decreased PKA activity in DMH GABAergic neurons of RII*β*‐KO mice. A) Unsupervised clustering of DMH GABAergic neuronal types represented in a t‐SNE plot. Cell‐type clusters are color‐coded. B) Expression patterns of PKA subunits in DMH GABAergic neurons of RII*β*‐KO and WT mice. C) GO analysis of DEGs in GABAergic neurons between RII*β*‐KO and WT mice. D) Diagram of the mechanisms underlying the decreased PKA activity of DMH GABAergic neurons in RII*β*‐KO mice.

To further characterize the expression patterns of PKA subunits in DMH GABAergic neurons of RII*β*‐KO and WT mice, we unsupervised clustering of DMH GABAergic neuronal types and represented in a t‐SNE plot (**Figure** **5**A). At the mRNA level, RII*β* deficiency led to approximately a 50% increase in the level of *Prkar1a* in GABAergic neurons, while the levels of the *Prkar1b*, *Prkar2a*, *Prkaca*, and *Prkacb* were reduced when compared with controls (Figure 5B; Figure S8A,C,D,E, Supporting Information). These findings validated that RII*β* deficiency decreases the level of catalytic subunits and thus reduces the overall PKA activity in DMH GABAergic neurons.

To decipher the molecular characteristics of GABAergic neurons in the DMH of RII*β*‐KO mice, we analyzed the DEGs for enrichment among Kyoto Encyclopedia of Genes and GO terms. GO analysis demonstrated a decreased PKA activity in DMH GABAergic neurons of RII*β*‐KO mice (Figure 5C). Both up‐ and downregulated DEGs were closely related to PKA activity, neurotransmitter transport and ion homeostasis (Figure S8A, Supporting Information). Additionally, correlation analysis demonstrated a close positive correlation between PKA activity and GABA transport (Figure S8B, Supporting Information) or PKA activity and glutamatergic activity (Figure S8F, Supporting Information). Collectively, these single‐cell sequencing data indicate that the RII*β* deficiency may result in a decreased PKA activity and increased neuronal excitability in the DMH GABAergic neurons (Figure 5D; Figure S8G, Supporting Information).

### 2.6 Inhibition of PKA Activity in DMH GABAergic Neurons Promotes WAT Browning and Reduces Adiposity

**Figure 6:**
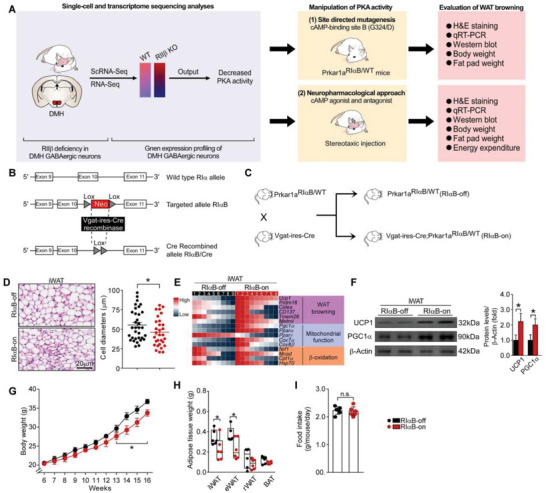
Inhibition of PKA activity in DMH GABAergic neurons by site‐directed mutagenesis promotes WAT browning and reduces adiposity. A) Schematic illustration of experiments. B) Strategy for generation of RI*α*B‐on mice. C) Breeding strategy for generation of Vgat‐ires‐Cre; Prkar1a^RI*α*B/WT^ mice (RI*α*B‐on). D) Representative images of H&E staining of iWAT and the size profiling of adipocytes from iWAT. Scale bar indicates 20 µm. E) Heatmap shows mRNA levels of the WAT browning associated genes in iWAT. F) Representative immunoblots of UCP1, PGC1*α*, and *β*‐Actin from iWAT, and the quantified ratio of UCP1/*β*‐Actin, PGC1*α*/*β*‐Actin. G) Body weight. H) Fat‐pad weight. I) Food intake. RI*α*B‐on *n* = 9; RI*α*B‐off *n* = 9. Values show mean ± SEM. Student's *t*‐test was used for analysis of the data in (D), (F), (H), and (I). One‐way ANOVA with Tukey's post hoc test was used for analysis of the data in (G). *p < 0.05.

The single‐cell sequencing and transcriptome sequencing data reveal a decreased PKA activity in DMH GABAergic neurons of RII*β*‐KO mice (**Figure** **6**A). To determine whether the PKA and GABAergic function‐associated DEGs in hypothalamic GABAergic neurons is associated with the adipose browning phenotypes, we used a mouse line in which PKA activity can be inhibited in certain cell types, and we also used neuropharmacological approach to specifically activate or inhibit PKA activity (Figure 6A). The mouse line carries a mutant Prkar1a allele encoding a glycine to aspartate substitution at position 324 in the carboxy‐terminal cAMP‐binding domain (site B).^[^ ^2^, ^6^, ^20^ ^]^ This mutation produces a dominant‐negative RI*α* regulatory subunit (RI*α*B) and leads to a potent inhibition of PKA activity (Figure 6B,C). As expected, the reduction of PKA activity in hypothalamic GABAergic neurons induced adipose browning, lowered body weight (final weight mean ± SEM: RI*α*B‐on, 34.8 ± 0.3 g; RI*α*B‐off, 36.5 ± 0.2 g), and reduced fat‐pad mass (iWAT and eWAT) (iWAT weight mean ± SEM: RI*α*B‐on, 0.32 ± 0.01 g; RI*α*B‐off, 0.42 ± 0.02 g; eWAT weight mean ± SEM: RI*α*B‐on, 0.34 ± 0.01 g; RI*α*B‐off, 0.44 ± 0.02 g), but did not alter the food intake (Figure 6D–I).

The neuropharmacological studies unravel that stereotaxic injection of PKA‐specific inhibitory cAMP analog (Rp‐cAMP) into DMH decreased the PKA activity as indicated by decreased phosphorylated protein levels of CREB and PKA substrates (Figure S9A–C, Supporting Information). In contrast, PKA activity was raised by treatment with the cAMP analog Sp‑cAMP (Figure S9C, Supporting Information). The inhibition of PKA activity in DMH induced WAT browning, lowered body weight, reduced fat‐pad weight, and elevated EE; and vice versa (Figure S9D–H,J, Supporting Information). Rp‐cAMP or Sp‐cAMP has no effect on food intake (Figure S9I, Supporting Information). These results validate that PKA activity in these hypothalamic GABAergic neurons is implicated in the central control of WAT browning.

### 2.7 Schematic Diagram of Mechanism Underlying RII*β*‐PKA in DMH GABAergic Neurons Regulates WAT Browning

**Figure 7:**
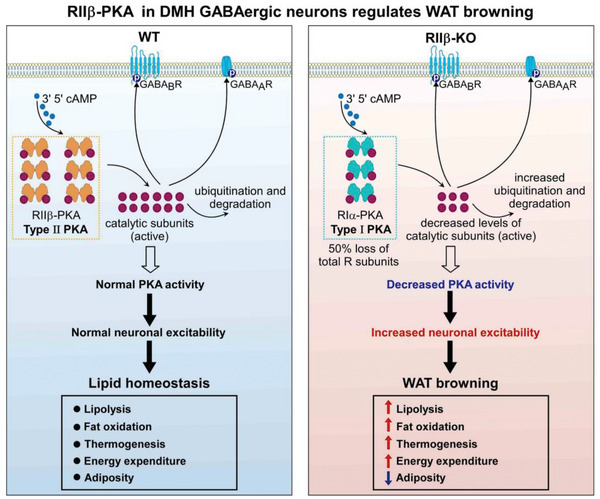
Schematic diagram of mechanism underlying RII*β*‐PKA in DMH GABAergic neurons regulates adipose browning.

Based on our previous studies and current work, we proposed a simple model describing the dynamic changes in PKA activity in DMH GABAergic neurons related to the central regulation of WAT browning, and illustrated in **Figure** **7**. In wild‐type DMH GABAergic neurons, the RII*β* subunits preferentially associate with C subunits to form Type‐II PKA. In RII*β* mutant DMH GABAergic neurons, the absence of the RII*β* results in a compensatory increase of RI*α* subunits and thus forms Type‐I PKA. This biochemical adaptation provides a very effective mechanism for regulating the ratio of Type‐II to Type‐I holoenzyme, and also for maintaining the basal PKA activity when RII*β* subunit is absent. Notably, although there is a compensatory increase of RI*α* subunits, it was calculated that there is a ∼50% loss of R subunits overall as measured by total cAMP‐binding capacity.^[^ ^2^, ^7^ ^]^ Additionally, lower affinity interaction between RI*α* and C*β* results in increased degradation of the free catalytic subunit.^[^ ^5^, ^7^, ^21^ ^]^ Thus, the PKA activity in DMH GABAergic neurons was reduced in RII*β*‐KO mice. This reduction of PKA activity may decrease the phosphorylation level of gamma‐aminobutyric acid type A receptor (GABA_A_R) and gamma‐aminobutyric acid type B receptor (GABA_B_R),^[^ ^20^, ^22^ ^]^ and thus increase the neuronal excitability of DMH GABAergic neurons. It is well recognized that sympathetic signals emanating from the DMH to adipose tissue play a central role in the regulation of WAT browning.^[^ ^14^, ^23^ ^]^ Therefore, the enhanced sympathetic outflow from DMH to the WAT contributes to the reduced adiposity of RII*β*‐KO mice by promoting adipose browning.

## 3 Discussion

Obesity is a complex, recurrent, and progressive chronic disease, defined as excessive fat accumulation.^[^ ^24^ ^]^ Obesity causes many complications including type 2 diabetes, hypertension, cardiovascular disease, and certain types of cancer.^[^ ^24^, ^25^ ^]^ According to the World Health Organization (WHO) global estimates, one out of five adults worldwide will be obese by 2025.^[^ ^26^ ^]^ In fact, more than 1.9 billion adults (39%) of 18 years and older were overweight; and over 650 million (13%) of these were obese.^[^ ^27^ ^]^ The stimulation of WAT browning has been considered a potential approach to treat obesity and other metabolic diseases.^[^ ^25^, ^28^ ^]^

In the present study, we show that RII*β*‐KO mice exhibit robust WAT browning phenotypes. We specifically reexpressed RII*β* in DMH GABAergic neurons, and this reexpression normalized the adipose browning and the lean phenotypes of RII*β*‐KO mice. Emerging evidence also shows that activation of leptin receptor‐expressing neurons within the DMH elevates EE and reduces adiposity.^[^ ^29^ ^]^ Disruption of leptin signaling in prolactin‐releasing peptide (PrRP) neurons in the DMH blocks the thermogenic response to leptin and causes obesity.^[^ ^30^ ^]^ Inhibition of NPY in DMH GABAergic neurons by AAV‐mediated RNAi promotes the development of beige adipocytes through sympathetic‐adipose connection.^[^ ^17^ ^]^ These observations have implicated the DMH in the central regulation of WAT browning, and highlight that RII*β*‐PKA in DMH GABAergic neurons may be a key signal transduction factor for governing the WAT browning and the whole‐body fat homeostasis.

It is reported that the increased neuronal excitability in the DMH leads to increased thermogenesis and elevated EE.^[^ ^17^, ^31^ ^]^ We observed that the excitability of the DMH GABAergic neurons was increased in RII*β*‐KO mice. The cAMP‐PKA system regulates neuronal excitability.^[^ ^32^ ^]^ Several lines of evidence show that knockout of RII*β*, RI*β* or C*β* subunit of PKA alters the PKA activity, resulting in decreased neuronal excitability, suppressed synaptic plasticity, and a defect of long‐term potentiation (LTP) in the hippocampal neurons.^[^ ^33^ ^]^ In this study, we observed that the frequency of action potentials and the mean input resistance in DMH GABAergic neurons of RII*β*‐KO mice were enhanced, demonstrating increased neuronal excitability. In addition, our single‐cell sequencing analysis also revealed that several unique clusters of DMH GABAergic neurons were activated, indicated by the increased expression levels of Fosb, a marker of neuronal activation. Moreover, our chemogenetic and optogenetic studies further confirmed this mechanism that the activation of DMH GABAergic neurons led to remarkable adipose browning and reduced fat accumulation.

RII*β* subunits are the principal PKA regulatory subunits in both the hypothalamus and BAT.^[^ ^2^, ^3^ ^]^ RII*β* subunits are predominately localized in neurons and are rarely detectable in glial cells.^[^ ^33^, ^34^ ^]^ In hypothalamic neurons, knockout of RII*β* gene causes decreased protein levels of the C subunits (including C*α*, C*β*1, and C*β*2) and the RII*α* subunit of PKA; and a slight but non‐significant increase in the protein level of RI*α*.^[^ ^2a^ ^]^ These alterations lead to an enzymatic subtype‐switch from Type‐II PKA to Type‐I PKA in hypothalamic neurons.^[^ ^2a^ ^]^

The RII*β*‐PKA (Type‐II PKA) is localized to the dendrites by interaction with A‐kinase anchor proteins (AKAPs), which confers a strong ability on RII*β*‐PKA to regulate the phosphorylation level of ion channels and receptors of neurotransmitters. Whereas, the RI*α*‐PKA and RI*β*‐PKA (type‐I PKA) have reduced affinity for many AKAPs like AKAP5 and thus are localized in the cytoplasm.^[^ ^20^ ^]^ Physiologically, the C subunits preferentially bind to the RII*β* subunits until the RII*β* binding capacity is saturated. Therefore, in the absence of RII*β*, the concentration of free C subunits is elevated, which in turn causes the increased RI*α* subunits to partially stabilize these free C subunits from proteolysis.^[^ ^3^, ^6^ ^]^ This compensatory biochemical response leads to a conversion of Type‐II PKA to Type‐I PKA, and a decreased overall PKA activity.^[^ ^1^, ^3^, ^7^ ^]^ Previous studies have shown a decreased protein level of both the phosphorylated PKA substrates and the CREB in the hypothalamus of RII*β*‐KO mice, reflecting a decreased PKA activity in hypothalamic neurons of RII*β*‐KO mice.^[^ ^2^, ^33^ ^]^ In agreement with these previous studies, the western analysis in our current work also showed a reduced level of phosphorylated PKA substrates and CREB in the DMH of RII*β*‐KO mice, demonstrating a decreased PKA activity in the DMH of RII*β*‐KO mice. Similar to the finding of previous study,^[^ ^2^, ^33^ ^]^ our single‐cell RNA‐sequencing analysis also showed an increased expression level of RI*α* and a reduced transcriptional level of C*β*, indicating the compensatory response of RI*α* subunit as well as the decreased PKA catalytic capability in DMH GABAergic neurons of RII*β*‐KO mice. Taken together, these results demonstrate that the conversion of type‐II PKA to type‐I PKA in GABAergic neurons of the DMH may contribute to the generation of the adipose browning and other healthy metabolic phenotypes; and highlight the important role of PKA in the central regulation of WAT browning.

The PKA regulates the GABAR channel conductance.^[^ ^35^ ^]^ The release of GABA from GABAergic neurons is also largely depending on the strength of the PKA activation level.^[^ ^36^ ^]^ The RII*β*‐PKA is localized to the dendrites, while the RI*α*‐PKA is localized in the cytosoma.^[^ ^20^ ^]^ Thus, the subcellular relocalization of PKA caused by RII*β* gene knockout results in an underphosphorylated status of the ion channels in dendrites on the membrane of neurons.^[^ ^20^ ^]^ For example, relocation of PKA to cytosoma causes a decreased phosphorylation of ion channels, and thus desensitizes GABA_A_R and GABA_B_R,^[^ ^22^, ^37^ ^]^ leading to disinhibition of GABAergic neurons.^[^ ^38^ ^]^ Nevertheless, although GABA receptor subunits might be the main targets of PKA signaling, we still cannot rule out the possibility that other receptors, such as glycine receptor or NMDA receptor, could also be phosphorylated by PKA and then directly or indirectly regulate neuronal excitability. Future work using heterologous expression systems for GABA subunits will be helpful to determine whether GABA receptors are the main targets that account for the increased excitability of DMH GABAergic neurons in RII*β*‐KO mice. Together, these findings suggest that the subtype‐conversion of PKA may cause increased neuronal excitability of GABAergic neurons in the DMH. The RII*β*‐PKA may serve as a key factor in the regulation of intrinsic excitabilities of GABAergic neurons. The deficiency of RII*β* may lead to increased neuronal excitability of the GABAergic neurons in the DMH, which may underlie the WAT browning and lean phenotypes of RII*β*‐KO mice.

To confirm whether the decreased PKA activity caused by this enzymatic subtype conversion in DMH GABAergic neurons are essential for eliciting WAT browning, we utilized a dominant‐negative PKA mutant mouse line, allowing selective expression of a dominant‐negative PKA subunit allele (RI*α*B) to suppress PKA activity and CREB phosphorylation in specific cells,^[^ ^2^, ^20^ ^]^ and we also used pharmacological approach to specifically inhibit PKA activity of DMH. In these studies, we found that the inhibition of PKA activity in the hypothalamic GABAergic neurons led to WAT browning and lowered fat accumulation. Overall, these observations point out that PKA activity in the GABAergic neurons of the DMH may be a fundamental factor for the central regulation of WAT browning; and RII*β*‐PKA in these neurons may be a potential pharmacological target for effectively promoting WAT browning.

## 4 Conclusion

In summary, this study reveals that adipose browning contributes to the healthy lean phenotypes of RII*β*‐KO mice. The GABAergic neurons in the DMH may be important neuronal populations that predominantly regulate adipose browning. The enzymatic subtype‐conversion of PKA induced by RII*β* gene knockout may be a crucial event for eliciting WAT browning of RII*β*‐KO mice. Targeting RII*β*‐PKA in the GABAergic neurons of the DMH via biomedical approaches may offer a clinically useful way to promote adipose browning for the treatment of obesity or metabolic comorbidities.

## 5 Experimental Section

### Mice and Animal Care

Mice were housed at 22 ± 1 °C with a 12‐h light/dark cycle. Standard mouse chow and water were freely available except where otherwise indicated. All procedures were approved by the Institutional Care and Use Committee of the Peking University Health Science Center (approval number: LA2019340). All animals were sex‐ and age‐matched, and littermates were used, as indicated in the figures. Animals were allocated to their experimental group according to their genotypes. RII*β*‐KO (RII*β* ^−/−^) mice, RII*β* ^lox/lox^ mice, and Prkar1aR1*α*B/WT mice were a kind gift from G. Stanley McKnight of University of Washington School of Medicine. To minimize the occurrence of germ‐line recombination, heterozygous Vgat‐RII*β* knocked (Vgat‐ires‐Cre; RII*β* ^+/−^) mice were crossed with RII*β* ^lox/lox^ mice to generate heterozygous RII*β* ^Vgat‐CRE^ mice (Vgat‐ires‐Cre; RII*β* ^lox/+^). Vgat‐ires‐Cre; Prkar1aR1*α*B/WT mice were obtained by crossing heterozygous Vgat‐ires‐Cre transgenic mice to Prkar1aR1*α*B/WT mice. This study referred to the Cre‐negative Prkar1aR1*α*B/WT as RI*α*B‐off and the Vgat‐ires‐Cre; Prkar1aR1*α*B/WT as RI*α*B‐on. Genotyping of mice carrying Vgat‐Cre, mutated RII*β*, lox RII*β* allele, and the Prkar1aR1*α*B/WT were performed as previously described.^[^ ^2^, ^6^ ^]^ During all procedures of experiments, the number of animals and their suffering by treatments were minimized.

### Hematoxylin and Eosin Staining

Animals were sacrificed and fats were immediately dissected and fixed in 4% paraformaldehyde solution for 48 h followed by cryopreservation in 25% sucrose solution (wt/vol) overnight and subsequent freezing in OCT compound (Tissue‐Tek). Samples were stored in optimal cutting temperature compound (OCT) for frozen. Samples were sectioned, and H&E stained. The cell size was calculated by Image J.

### Quantitative Real‐Time PCR

RNA was extracted from the tissues using TransZol Up reagent (ET111‐01, TransGen Biotech), followed by reverse transcription using a reverse transcription kit (AT311‐03, TransGen Biotech). cDNAs were processed for real‐time PCR using SYBR Green mix (AQ141‐04, TransGen Biotech) with specific primers on a StepOnePlus real‐time PCR System (Roche). All data were normalized with GAPDH.

### Total Protein Extraction and Western Blotting

Proteins were extracted from iWAT or DMH using a RIPA lysis buffer containing 0.5% NP‐40, 0.1% sodium deoxycholate, 150 mm NaCl, 50 mm Tris–HCl (pH 7.4), phosphatase inhibitors (B15002, Bimake), and protease inhibitor cocktail (B14002, Bimake). Following 5 min of homogenization, lysates were centrifuged at 11 250 r for 15 min at 4 °C. Supernatants from the fat pads were used as protein extracts. The concentration of each sample was calculated by the BCA method and an equal amount of protein from each sample was added an equal amount of protein loading buffer (This buffer should contain 5% *β*‐mercaptoethanol (vol/vol)), and denatured by boiling at 100 °C for 5 min. Equal amounts of proteins were separated by 10% SDS‐PAGE, transferred to NC membranes. The membranes were blocked for 2 h in 5% skim milk. The membranes were then incubated with the primary antibody in 5% BSA‐TSBT at 4 °C. After overnight incubation, the membrane was washed three times in TBST for 15 min, followed by incubation with a secondary antibody in TBST with 5% skim milk for 2 h at room temperature. Following three cycles of 15 min washes with 1× TBST, the membranes were developed using a chemiluminescence assay. Intensities of the protein bands were quantified by ImageJ software. The antibodies used in this study include anti‐UCP1 (Abcam, ab10983; Bioss, bs‐1925R), Anti‐Pgc1*α* (Santa Cruz Biotechnology, sc‐13067), anti‐CREB (Cell Signaling Technology, 9197S), Anti‐pCREB (Cell Signaling Technology, 9198S), Anti‐phospho‐PKA substrates (Cell Signaling Technology, 9621S), anti‐GAPDH (Sigma‐Aldrich, G8795), Anti‐*β*‐Actin (Sigma‐Aldrich, A5316), Anti‐TH (Abcam, ab152), Anti‐Tubulin (Cell Signaling Technology, 2148S), Anti‐Rat IgG(H+L) (Biodragon, BF03008), and Anti‐Mouse IgG(H+L) (Biodragon, BF03001).

### Metabolic Chamber

The mice were placed in metabolic chambers with fresh food and water provided every day to acclimate for 24 hr. On day 7, before dark cycle, the authors started to monitor the oxygen consumption (VO_2_), carbon dioxide production (VCO_2_), respiratory‐exchange‐ratio (RER), energy expenditure (EE), and motor activity for 24 h (LE1305 Physiocage 00; LE405 O_2_/CO_2_ Analyzer; LE400 Air Supply and Switching). Metabolism v2.2.01 was used to analyze the data. These metabolic parameters were adjusted for mouse adiposity.

### Brain Slice Preparation for Microdissection and Single‐Cell Dissociation

Single‐cell dissociations for the scRNA‐seq experiments were performed on DMH tissue from mouse brain slices. The ScRNA‐Seq was as independent experiment. For scRNA‐seq experiment, DMH tissues were obtained from five male RII*β*‐KO mice (pooled) and five male WT mice (pooled) (8 weeks old). All mice were killed by rapid decapitation following isoflurane anesthesia, within the same time (morning, 8:00–11:00). Two to three slices were obtained from each animal that approximately corresponded to mouse brain atlas images representing the distance from bregma −1.34, −1.84 and −2.34 mm. All microdissections were confined to the DMH, but in some cases may also include portions of the VMH, as indicated in Figure 4A. Microdissected tissue punches were kept in dissociation solution (1 mm L‐gystein, 0.5 mm Na_2_EDTA, DNase‐I 100 U mL, ovomucoid inhibitor 10 mg mL^−1^, 10 mg mL^−1^ BSA) on ice until trituration. Tissues were enzyme‐treated for ∼30 min at 37 °C using papain (20 U mL; Worthington) in a dissociation solution containing the following components: 1 mm L‐gystein, 0.5 mm Na_2_EDTA and DNase‐I (100 U mL; Sigma). Single‐cell suspensions were passed through 60 µm nylon mesh filters to remove any cell aggregates, and cells were harvested by centrifugation and resuspended in EBSS (M&C #CC029) containing the following components: 0.05 mm DL‐AP5, 0.05 mm kynurenic acid and 150 mm D‐trehalose, and kept on ice until single‐cell capture.

### scRNA‐seq Cell Capture and Sequencing

The viability of each single‐cell suspension was assessed using a LUNA‐FLTM Dual Fluorescence Cell Counter. Suspensions of dissociated cells were filtered through 20 µm nylon mesh filters to remove cell aggregates and large debris and were loaded onto independent single channels of a Chromium Controller (10× Genomics) single‐cell platform. Briefly, ∼10 000 single cells were loaded for capture using a Chromium Single Cell 3′ Reagent kit, v2 Chemistry (10× Genomics). Following capture and lysis, complementary DNA was synthesized and amplified (14 cycles) as per the manufacturer's protocol (10× Genomics). The amplified cDNA was used to construct an Illumina sequencing library and sequenced on a single lane of a HiSeq 4000 (Illumina). For FASTQ generation and alignments, Illumina basecall files (*.bcl) were converted to FASTQs using Cell Ranger v.1.3 (10× Genomics), which uses bcl2fastq v.2.17.1.14. FASTQ files were then aligned to mm10 genome and transcriptome using the Cell Ranger v.1.3 pipeline, which generates a “gene versus cell” expression matrix.

### scRNA‐seq Analysis

For filtering and unsupervised clustering, the gene expression matrix from Cell Ranger was used for downstream analysis (using R 3.6.1). Of the initial 12124 cells (5755 WT mice and 6369 RII*β*KO mice), cells with less than 500 UMIs or >40% of mitochondrial reads were discarded. Genes with at least 2 counts in 5 cells were used for downstream analysis. Gene expression of the remaining cells was normalized by the total number of transcripts detected in each cell and multiplied by the median transcript count. The normalized expression was log_2_‐transformed after addition of a pseudocount. The top 1000 genes with the most variance were identified based on their mean expression in the population and dispersion (variance/mean expression). Genes were binned into 50 different bins based on their mean expression and dispersion scaled with respect to the median dispersion in each bin. These genes were used to reduce the dimensions of the dataset using Barnes Hut t‐SNE with default parameters. Cells were clustered in t‐SNE space using SEURAT. In each case, this was through an iterative process, testing different parameters and visualizing ellipse thresholds to ensure that clusters were optimally resolved into distinct units in t‐SNE space. The calculation for the top 1000 genes, dimensionality reduction and clustering were performed on a combined gene expression matrix, which led to a single representation of cells in t‐SNE space.

To differentiate neuronal and non‐neuronal clusters, the entire gene versus cell matrix was filtered and clustered as described above. To classify a cluster as a neuron or a non‐neuron cluster, the median expression of the known neuron markers Snap25, Syp, Tubb3, and Elavl2 were aggregated for each cluster. The median expression of these genes was aggregated in each cluster and a cluster was classified as high (neuronal) or low (non‐neuronal) expression using a simple Gaussian mixture model. Every cell was then classified as a neuron or non‐neuron based on their cluster membership. Subsequent clustering of non‐neuronal and neuronal populations was based on this classification. For the classification of GABAergic and glutamatergic clusters, clusters classified as neurons were combined and re‐clustered as described above, which yielded 9 clusters. Clusters were classified as GABAergic if the median expression of Slc32a1 is greater than Slc17a6 in each cluster and glutamatergic if the median expression of Slc17a6 is greater than Slc32a1 (Figure 3). Genes differentially expressed in a given cluster were computed using edgeR.

### Bulk RNA‐seq and Analysis

The Bulk RNA‐seq was an independent experiment. For bulk RNAseq experiment, DMH tissues were obtained from five male RII*β*‐KO mice (pooled) and five male WT mice (pooled) (8 weeks old). A total amount of 3 µg RNA per sample was used as input material for the RNA sample preparations. Sequencing libraries were generated using NEBNext Ultra RNA Library Prep Kit for Illumina (NEB, USA) following manufacturer's recommendations and index codes were added to attribute sequences to each sample. Briefly, mRNA was purified from total RNA using poly‐T oligo‐attached magnetic beads. Fragmentation was carried out using divalent cations under elevated temperature in NEBNext First Strand Synthesis Reaction Buffer (5×). First strand cDNA was synthesized using random hexamer primer and M‐MuLV Reverse Transcriptase (RNase H). Second strand cDNA synthesis was subsequently performed using DNA Polymerase I and RNase H. Remaining overhangs were converted into blunt ends via exonuclease/polymerase activities. After adenylation of 3′ ends of DNA fragments, NEBNext Adaptor with hairpin loop structure were ligated to prepare for hybridization. In order to select cDNA fragments of preferentially ∼150–200 bp in length, the library fragments were purified with AMPure XP system (Beckman Coulter, Beverly, USA). Then 3 µL USER Enzyme (NEB, USA) was used with size‐selected, adaptor‐ligated cDNA at 37 °C for 15 min followed by 5 min at 95 °C before PCR. Then PCR was performed with Phusion High‐Fidelity DNA polymerase, Universal PCR primers and Index (X) Primer. At last, PCR products were purified (AMPure XP system) and library quality was assessed on the Agilent Bioanalyzer 2100 system.

The clustering of the index‐coded samples was performed on a cBot Cluster Generation System using TruSeq PE Cluster Kit v3‐cBot‐HS (Illumia) according to the manufacturer's instructions. After cluster generation, the library preparations were sequenced on an Illumina Hiseq 2000/2500 platform and 100 /50 bp single‐end reads were generated.

Differential expression analysis of two conditions/groups (five biological replicates per condition) was performed using the DESeq R package (1.10.1). DESeq provide statistical routines for determining differential expression in digital gene expression data using a model based on the negative binomial distribution. The resulting *p*‐values were adjusted using the Benjamini and Hochberg's approach for controlling the false discovery rate. Genes differentially expressed over 1.5 folds between the compared groups, and with an adjusted *p*‐value <0.05 found by DESeq were assigned as differentially expressed.

### Stereotaxic Injection of AAV

Mice were anesthetized with isoflurane and placed in a stereotaxic apparatus. AAV2/9 was chosen for precisely targeting small brain region as previously described.^[^ ^39^ ^]^ AAV2/9‐Vgat1‐GFP, AAV2/9‐Vgat1‐Cre‐GFP, AAV2/9‐DIO‐hM_3_Dq‐GFP, AAV2/9‐DIO‐hM_4_Di‐GFP, AAV2/9‐DIO‐GFP, or AAV2/9‐DIO‐ChR2‐GFP (2 × 10^12^ viral particles) were bilaterally injected into DMH (coordinates, bregma: anterior‐posterior, −1.55 mm; dorsal‐ventral, −5.30 mm; lateral, ± 0.35 mm, 0.2 µL per side) of the mice, followed by recovery and AAV expression for 2 weeks. For those administered with AAV2/9‐DIO‐hM_3_Dq‐GFP and AAV2/9‐DIO‐hM_4_Di‐GFP, mice received twice‐daily intraperitoneal injections of CNO (Sigma‐Aldrich) or saline (vehicle) for 7 days at room temperature. CNO was administered i.p. at a dosage of 0.3 mg kg^−1^ as previously described.^[^ ^40^ ^]^ For those mice administered with AAV2/9‐DIO‐GFP or AAV2/9‐DIO‐ChR2‐GFP, an optical fiber (200 µm core, NA 0.22) was inserted into the brain and the fiber tip was placed above the DMH. Following 2 weeks of recovery, mice were photostimulated for 5 min (470 nm, 20 Hz) every 3 h repeated over 14 days. Correct DMH AAV delivery was assessed by post hoc immunofluorescence analysis after AAV injection.^[^ ^18^ ^]^

### Brain Slice Preparation for Patch Clamp

Brain slices were prepared from mice about 8 weeks of age. Coronal slices of DMH (300 µm thick) were cut with Campden vibratome (7000smz, Campden Instruments, Loughborough, UK) in prechilled and carbogen‐saturated (95% O_2_ / 5% CO_2_) NMDG‐based solution (92 mm NMDG, 2.5 mm KCl, 1.25 mm NaH_2_PO_4_, 30 mm NaHCO_3_, 20 mm HEPES, 25 mm glucose, 2 mm thiourea, 5 mm Na‐ascorbate, 3 mm Na‐pyruvate, 0.5 mm CaCl_2_, and 10 mm MgSO_4_, pH 7.3–7.4). Then the slices were incubated in carbogen‐saturated ACSF (126 mm NaCl, 2.5 mm KCl, 1.2 mm MgCl_2_, 2.4 mm CaCl_2_, 1.2 mm NaH_2_PO_4_, 21.4 mm NaHCO_3_, 10 mm glucose) at room temperature for at least 1 h before recording. After that, the slices were transferred to a recording chamber perfused with ACSF at a flow rate of ∼ 2 mL min^−1^.

### Cell Attached Recording and Whole‐Cell Recordings

All electrophysiological recordings were performed on GFP labeled GABAergic neurons at room temperature with Multiclamp 700B amplifier (Molecular Device, USA). Signals were recorded and converted to digital data via 1440 A/D converter (Axon Instruments) with sampling at 20 kHz and filtering at 3 kHz. Cell attached recordings and whole‐cell recording were performed with standard borosilicate glass capillaries. Cell attached recording electrodes filled with internal solution (128 mm K‐gluconate, 10 mm HEPES, 1 mm EGTA, 10 mm KCl, 1 mm MgCl_2_, 0.3 mm CaCl_2_, 3 mm Mg‐ATP, and 0.3 mm Na‐GTP, pH 7.35 with KOH) with resistance 3–6 MΩ. After cell‐attached recording, cell‐attached recordings were converted to whole‐cell patch‐clamp recording to record resting membrane potential. Labeled neurons firing rates were measured with cell‐attached recordings in voltage‐clamp recording and whole‐cell current clamp recordings. Membrane potential was measured by whole‐cell current clamp recordings from labeled neurons in brain slices. Liquid junction potential correction was performed off‐line.

### Canula Implantation

To locally and specifically activate or inhibit PKA activity in DMH, brain infusion cannula was implanted in the DMH. After recovery for 7 days, mice were infused twice with 0.5 µL of artificial CSF (ACSF), 0.5 µL of 40 nmol of Rp‐cAMP (in ACSF, Santa Cruz, sc‐24010), or 40 nmol of Sp‐cAMP (in ACSF, Santa Cruz, sc‐201571) using a micro dialysis probe under briefly isoflurane anesthesia. The doses of Rp‐cAMP or Sp‐cAMP were based on studies showing that this amount of PKA‐inhibitor or activator infused into the CNS was able to modulate CREB phosphorylation.

### Statistical Analysis

Where indicated, data are expressed as mean ± standard error of means (SEM). Data distribution was assessed using the Kolmogorov‐Smirnov test. Statistical analysis was performed using SPSS (Windows version 26, IBM Analytics) or GraphPad Prism (Windows version 8.0, GraphPad Software), with a *p*‐value of less than 0.05 considered significant. Statistical significance was determined by the unpaired two‐tailed Student's *t*‐test in comparisons between two groups. For cases using one‐way ANOVA or two‐way ANOVA, the Tukey's multiple comparisons tests were performed for post hoc tests. *p* values were indicated with single asterisk (*p < 0.05) and double asterisks (**p < 0.01) on graphs. Sample sizes (n), statistical tests and *p* values are indicated in each figure legend.

## Conflict of Interest

The authors declare no conflict of interest.

## Author Contributions

B.W. performed the experiment, analyzed the data, made the figures, and wrote the paper. M.Z., Z.S., B.J., X.Y., C.Z., B.G., J.L., W.H., J.L., Y.Z., Y.H., and F.L. participated in experiments. W.Z., L.Q., W.Z., and J.L. edited the paper. R.Z. conceived the study, designed experiments, and wrote and edited the paper. All authors reviewed and approved the manuscript for submission.

## Supporting information

Supporting Information

## Data Availability

The data that support the findings of this study are available in the supplementary material of this article.
