# Supplementary material for: RIIβ‐PKA in GABAergic Neurons of Dorsal Median Hypothalamus Governs White Adipose Browning
Source: Adv Sci (Weinh). 2022 Dec 18;10(5):2205173. doi: 10.1002/advs.202205173 (PMC9929258; doi:10.1002/advs.202205173)
Supplement: Supplementary file 1 — Supporting Information [file ADVS-10-2205173-s001.pdf]

ADVANCED SCIENCE
Open Access

# Supporting Information

for *Adv. Sci.*, DOI 10.1002/advs.202205173

RIIβ-PKA in GABAergic Neurons of Dorsal Median Hypothalamus Governs White Adipose Browning

*Bingwei Wang, Miao Zhao, Zhijie Su, Baohua Jin, Xiaoning Yang, Chenyu Zhang, Bingbing Guo, Jiebo Li, Weili Hong, Jiarui Liu, Yun Zhao, Yujia Hou, Futing Lai, Wei Zhang, Lihua Qin, Weiguang Zhang, Jianyuan Luo and Ruimao Zheng**

**Supporting Information**

RIIβ-PKA in GABAergic Neurons of Dorsal Median Hypothalamus Governs White Adipose Browning

*Bingwei Wang, Miao Zhao, Zhijie Su, Baohua Jin, Xiaoning Yang, Chenyu Zhang, Bingbing Guo, Jiebo Li, Weili Hong, Jiarui Liu, Yun Zhao, Yujia Hou, Futing Lai, Wei Zhang, Lihua Qin, Weiguang Zhang, Jianyuan Luo, Ruimao Zheng**

**Table S1.** Primers Used in This Study.

| Primer | Forward Primer 5'-3' | Reverse Primer 5'-3' |
|---|---|---|
| Ucp1 | ACTGCCACACCTCCAGTCATT | CTTTGCCTCACTCAGGATTGG |
| Prdm16 | CAGCACGGTGAAGCCATTC | GCGTGCATCCGCTTGTG |
| Cidea | TGCTCTTCTGTATCGCCCAGT | GCCGTGTTAAGGAATCTGCTG |
| CD137 | CGTGCAGAACTCCTGTGATAAC | GTCCACCTATGCTGGAGAAGG |
| Tmem26 | ACCCTGTCATCCCACAGAG | TGTTTGGTGGAGTCCTAAGGTC |
| Metrnl | CTGGAGCAGGGAGGCTTATTT | GGACAACAAAGTCACTGGTACAG |
| Pgc1α | AGCCGTGACCACTGACAACGAG | GCTGCATGGTTCTGAGTGCTAAG |
| Pparα | GGGTACCACTACGGAGTTCACG | CAGACAGGCACTTGTGAAAACG |
| Pparγ | GTGCCAGTTTCGATCCGTAGA | GGCCAGCATCGTGTAGATGA |
| Cox7α | CAGCGTCATGGTCAGTCTGT | AGAAAACCGTGTGGCAGAGA |
| Cox8β | GAACCATGAAGCCAACGACT | GCGAAGTTCACAGTGGTTCC |
| Nrf1 | CAGCAACCCTGATGGCACCGTGTC | GGCCTCTGATGCTTGCGTCGTCTG |
| Mcad | ATGACGGAGCAGCCAATGAT | TCGTCACCCTTCTTCTCTGCTT |
| Cpt1α | TGGCATCATCACTGGTGTGTT | GTCTAGGGTCCGATTGATCTTTG |
| HSP70 | TGGTGCTGACGAAGATGAAG | AGGTCGAAGATGAGCACGTT |
| Gapdh | AGGTCGGTGTGAACGGATTTG | TGTAGACCATGTAGTTGAGGTCA |

## SUPPLEMENTAL FIGURE LEGENDS

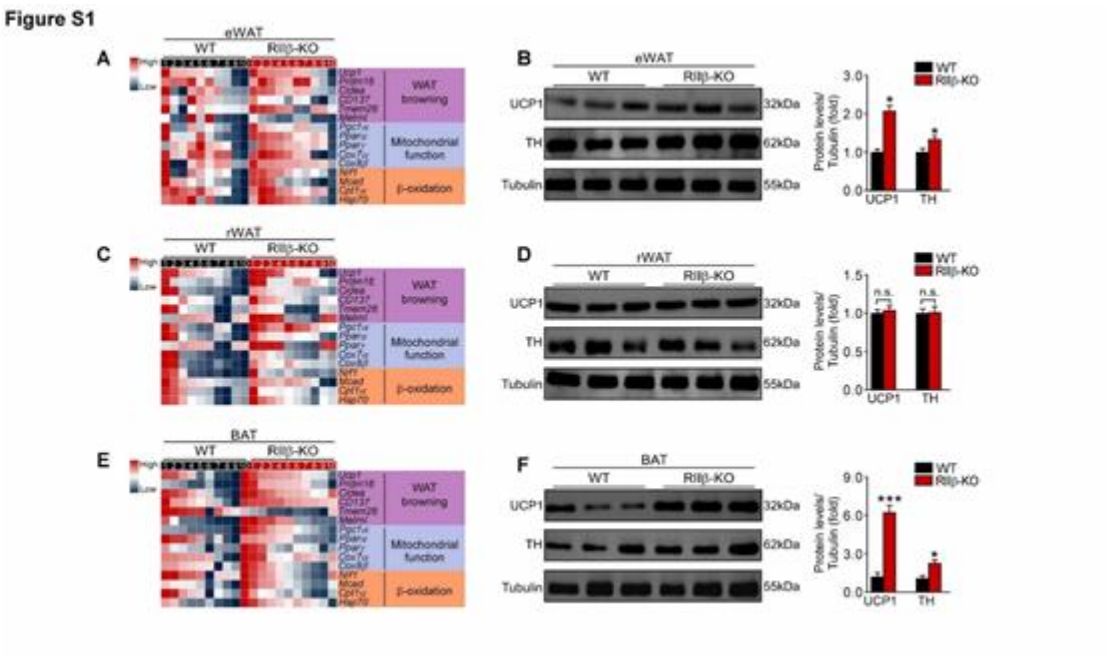


**Figure S1. mRNA and Protein Levels of Genes Associated with WAT browning in eWAT, rWAT and BAT.** (**A**) Heatmap shows mRNA levels of the WAT browning associated genes in eWAT. (**B**) Representative immunoblots of UCP1, TH and Tubulin from eWAT, and the quantified ratio of UCP1/Tubulin and TH/Tubulin. (**C**) Heatmap shows mRNA levels of the WAT browning associated genes in rWAT. (**D**) Representative immunoblots of UCP1, TH and Tubulin from rWAT, and the quantified ratio of UCP1/Tubulin and TH/Tubulin. (**E**) Heatmap shows mRNA levels of the WAT browning associated genes in BAT. (**F**) Representative immunoblots of UCP1, TH and Tubulin from BAT, and the quantified ratio of UCP1/Tubulin and TH/Tubulin. WT n = 10; RIIβ-KO n = 10. Values show mean ± SEM. Student's t-test was used for analysis of the data in (B), (D) and (F).

**Figure S2**

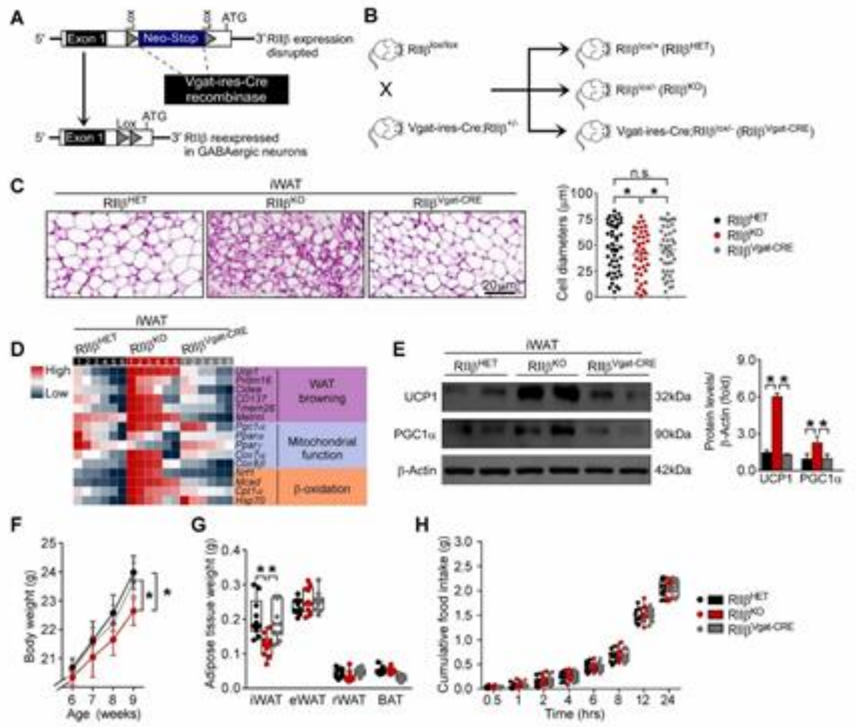


**Figure S2. Reexpression of RIIβ in GABAergic Neurons Abrogates WAT Browning** (**A**) Strategy for generation of Vgat-ires-Cre;RIIβ$^{lox/-}$ mice (RIIβ$^{Vgat\text{-}CRE}$ mice). (**B**) Breeding strategy for generation of RIIβ$^{Vgat\text{-}CRE}$ mice with GABAergic neurons-specific RIIβ reexpression. (**C**) Representative images of H&E staining of iWAT and the size profiling of adipocytes from iWAT. Scale bar indicates 20 μm. (**D**) Heatmap shows mRNA levels of the WAT browning associated genes in iWAT (RIIβ$^{HET}$ n = 6; RIIβ$^{KO}$ n = 6; RIIβ$^{Vgat\text{-}CRE}$ n = 6). (**E**) Representative immunoblots of UCP1, PGC1α and β-Actin from iWAT, and the quantified ratio of UCP1/β-Actin, PGC1α/β-Actin. (**F**) Body weight. (**G**) Fat-pad weight. (**H**) Cumulative food intake. RIIβ$^{HET}$ n = 10; RIIβ$^{KO}$ n = 10; RIIβ$^{Vgat\text{-}CRE}$ n = 10. Values show mean ± SEM. Two-way ANOVA with Tukey's post hoc test was used for analysis of the data in (C) and (E - H). *P < 0.05.

**Figure S3**

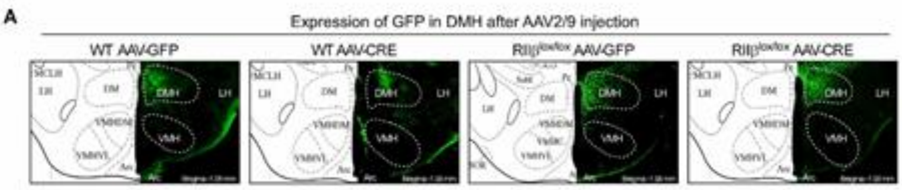

**Figure S3. Expression of GFP in DMH at week 7 after AAV2/9 injection. A**) Detailed AAV delivery sites and GFP-expressing GABAergic neurons in DMH. Mice were sacrificed at week 7 post-AAV injection. WT GFP n = 10; WT CRE n = 10; RIIβ$^{lox/lox}$ GFP n = 10; RIIβ$^{lox/lox}$ CRE n = 10.

**Figure S4**

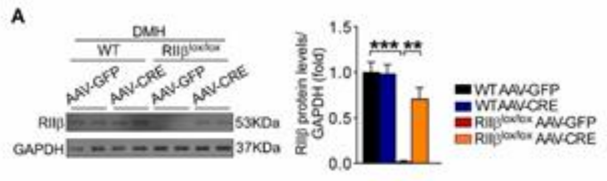


**Figure S4. Protein Level of Re-expressed RIIß Subunit in DMH. A**) Representative immunoblots of RIIβ and GAPDH from DMH, and the quantified ratio of RIIβ/GAPDH. WT GFP n = 10; WT CRE n = 10; RIIβ$^{lox/lox}$ GFP n = 10; RIIβ$^{lox/lox}$ CRE n = 10. Values show mean ± SEM. Two-way ANOVA with Tukey's post hoc test was used for analysis of the data. **P < 0.01 and ***P < 0.001.

**Figure S5**

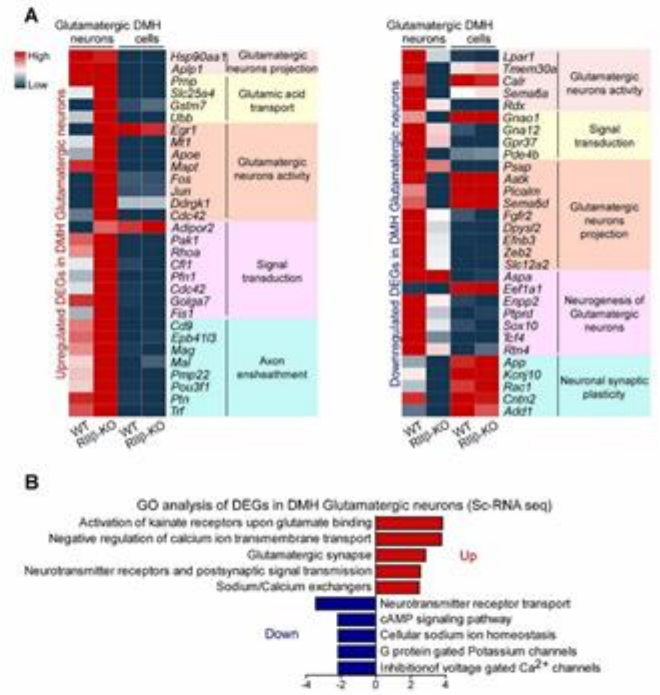


**Figure S5. Glutamatergic Function in DMH Glutamatergic Neurons of RIIβ-KO Mice** (**A**) Heatmap shows mRNA levels of the glutamatergic function associated genes in DMH at both single cell RNA sequencing (Glutamatergic neurons) and bulk RNA sequencing (DMH

cells) resolution. (**B**) GO analysis of DEGs in DMH Glutamatergic neurons (Sc-RNA seq) between RIIβ-KO and WT mice.

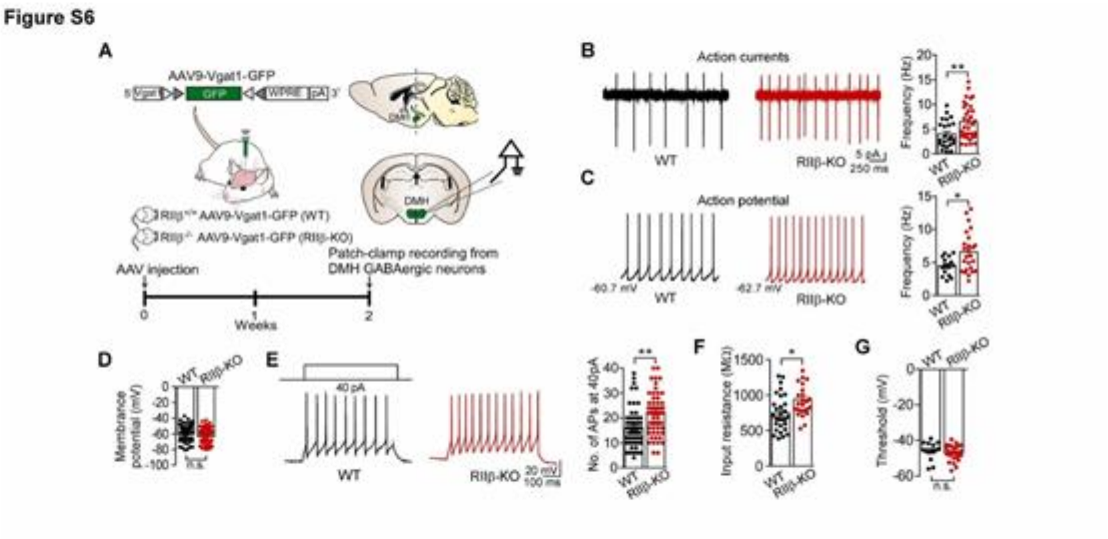


**Figure S6. Heightened Excitability of DMH GABAergic Neurons in RIIβ-KO Mice** (**A**) Schematic diagram of the experiment. (**B**) Representative cell-attached, current-clamp recording from DMH GABAergic neurons labelled by GFP fluorescence in the AAV-Vgat1-GFP injected mice. (**C**) Representative whole-cell, current-clamp recording from DMH GABAergic neurons labeled by GFP fluorescence in the AAV-Vgat1-GFP stereotaxically injected mice. (**D**) The resting membrane potentials of DMH GABAergic neurons. (**E**) Action potentials (APs) evoked by a depolarizing current step of 40 Pa in 500 ms. (**F**) The input resistance of DMH GABAergic neurons. (**G**) The resting threshold of DMH GABAergic neurons. All measures are from 10 mice per group. Values show mean ± SEM. Student's t-test was used for analysis of the data in (B - G). *P < 0.05 and **P < 0.01.

Figure S7

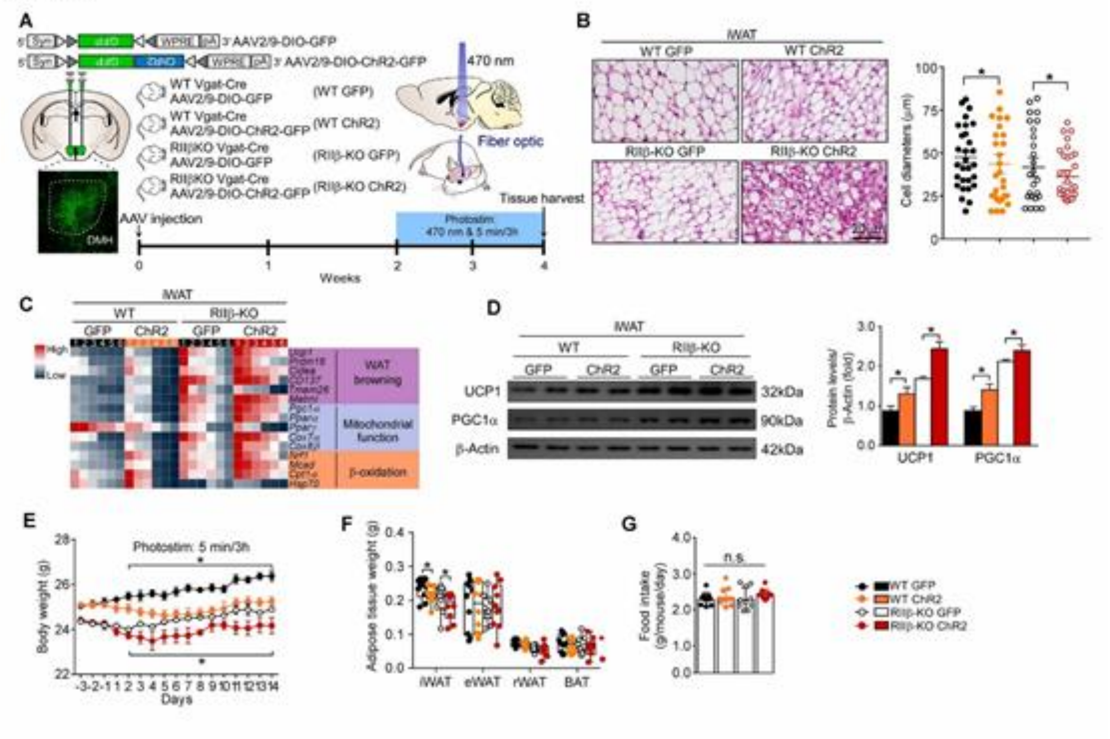


**Figure S7. Optogenetic Activation of DMH GABAergic Neurons Promotes WAT Browning and Reduces Adiposity** (**A**) Schematic diagram of the experiment and representative fluorescent image showing injection of AAV into DMH. (**B**) Representative images of H&E staining of iWAT and the size profiling of adipocytes from iWAT. Scale bar indicates 20 µm. (**C**) Heatmap shows mRNA levels of the adipose browning associated genes in iWAT. WT GFP n = 6; WT ChR2 n = 6; RIIβ-KO GFP n = 6; RIIβ-KO ChR2 n = 6. (**D**) Representative immunoblots of UCP1, PGC1α and β-Actin from iWAT, and the quantified ratio of UCP1/β-Actin, PGC1α/β-Actin. (**E**) Body weight. (**F**) Fat-pad weight. (**G**) Food intake. WT GFP n = 9; WT ChR2 n = 9; RIIβ-KO GFP n = 9; RIIβ-KO ChR2 n = 9. Values show mean ± SEM. Two-way ANOVA with Tukey's post hoc test was used for analysis of the data in (B) and (D - G). *P < 0.05 and **P < 0.01.

Figure S8

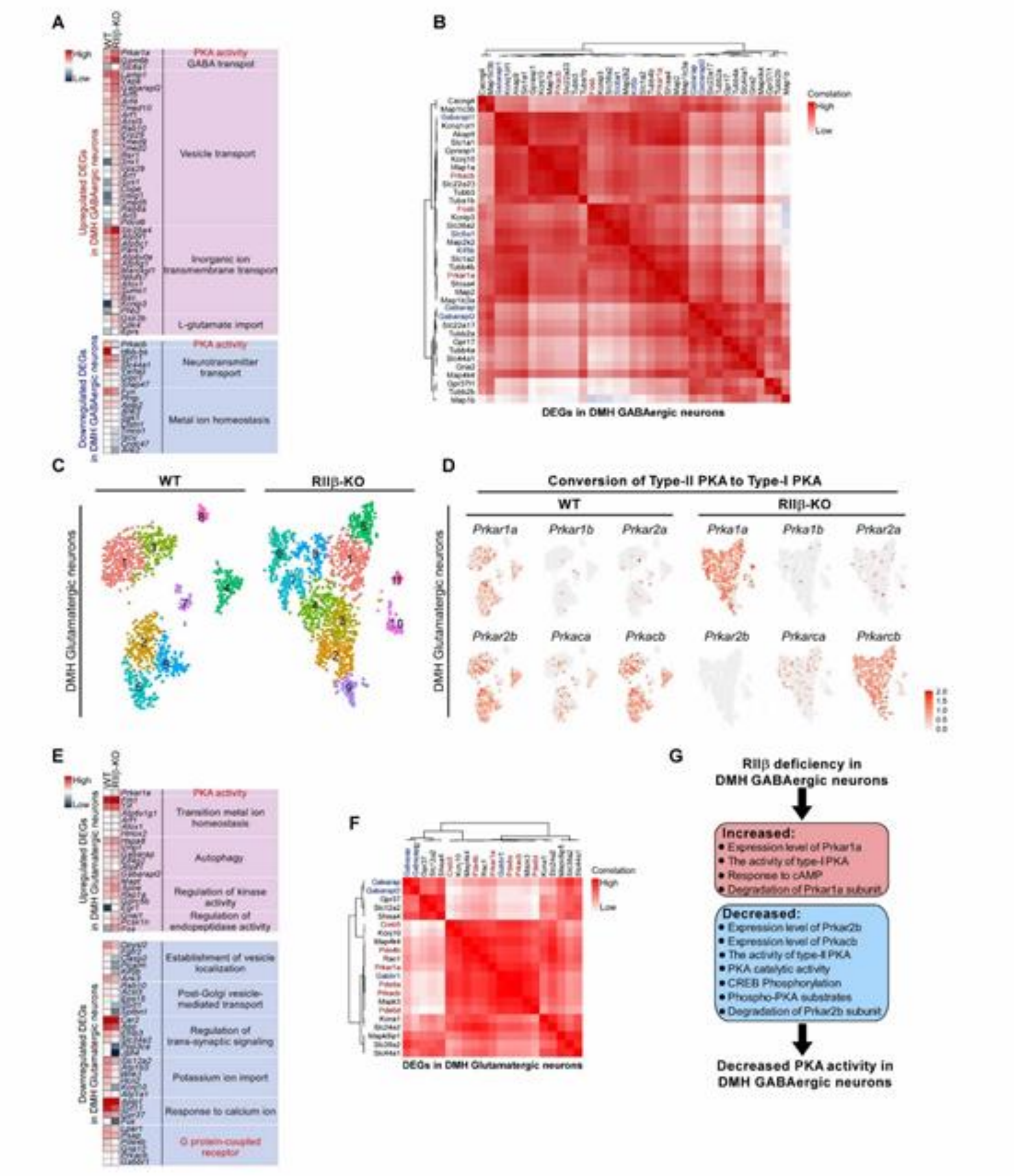


**Figure S8. Single-Cell Sequencing Analysis Reveals a Decreased PKA Activity in DMH GABAergic neurons of RIIβ-KO Mice** (**A**) Most significantly altered (based on P-value rank) genes (rows) in DMH GABAergic neurons of RIIβ-KO and WT mice (columns). Column-scaled z-scores computed with a Poisson mixed model are shown (FDR < 0.01, two-sided Wilcoxon rank-sum test). (**B**) Gene-gene correlation heatmap of the DEGs in DMH GABAergic neurons between RIIβ-KO and WT mice. (**C**) Unsupervised clustering of DMH Glutamatergic neuronal types represented in t-SNE plot. Cell-type clusters are color-coded.

(**D**) Expression patterns of PKA subunits in DMH Glutamatergic neurons of RIIβ-KO and WT mice. (**E**) Most significantly altered (based on P-value rank) genes (rows) in DMH Glutamatergic neurons in RIIβ-KO and WT mice (columns). Column-scaled z-scores computed with a Poisson mixed model are shown (FDR < 0.01, two-sided Wilcoxon rank-sum test). (**F**) Gene-gene correlation heatmap of the DEGs in DMH Glutamatergic neurons in RIIβ-KO and WT mice. (**G**) Schematic diagram showing the mechanism of the decreased PKA activity in DMH GABAergic neurons of RIIβ-KO mice.

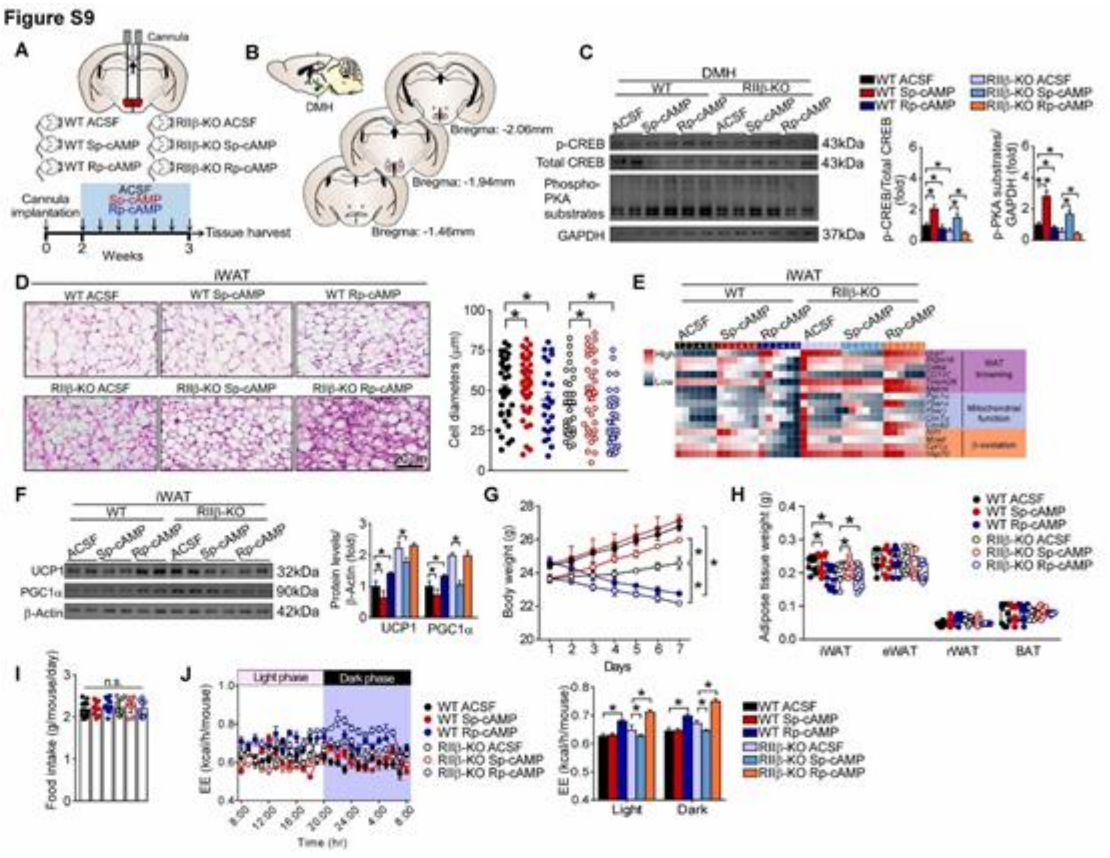


**Figure S9. Neuropharmacological Inhibition of PKA Activity in DMH Promotes WAT Browning and Reduces Adiposity** (**A**) Schematic diagram of the experiment. (**B**) Schematic representation of stereotaxic injection sites. (**C**) Representative immunoblots of p-CREB, CREB, Phospho-PKA substrates and GAPDH from DMH, and the quantified ratio of p-CREB/Total CREB, p-PKA substrates/GAPDH. WT ACSF n = 10; WT Sp-cAMP n = 10; WT Rp-cAMP n = 10; RIIβ-KO ACSF n = 10; RIIβ-KO Sp-cAMP n = 10; RIIβ-KO Rp-cAMP n = 10. (**D**) Representative images of H&E staining of iWAT and the size profiling

of adipocytes from iWAT. Scale bar indicates 20 μm. (**E**) Heatmap shows mRNA levels of the WAT browning associated genes in iWAT. WT ACSF n = 6; WT Sp-cAMP n = 6; WT Rp-cAMP n = 6; RIIβ-KO ACSF n = 6; RIIβ-KO Sp-cAMP n = 6; RIIβ-KO Rp-cAMP n = 6. (**F**) Representative immunoblots of UCP1, PGC1α and β-Actin from iWAT, and the quantified ratio of UCP1/β-Actin, PGC1α/β-Actin. (**G**) Body weight. (**H**) Fat-pad weight. (**I**) Food intake. WT ACSF n = 10; WT Sp-cAMP n = 10; WT Rp-cAMP n = 10; RIIβ-KO ACSF n = 10; RIIβ-KO Sp-cAMP n = 10; RIIβ-KO Rp-cAMP n = 10. (**J**) Energy expenditure (n = 5 per group). Values show mean ± SEM. Two-way ANOVA with Tukey's post hoc test was used for analysis of the data in (C), (D) and (F - J). *P < 0.05.
